# Supplementary material for: Enhancing Care Through a Virtual Canadian Community of Practice for Managing Immune-Related Adverse Events
Source: Curr Oncol. 2025 Feb 27;32(3):140. doi: 10.3390/curroncol32030140 (PMC11941491; doi:10.3390/curroncol32030140)
Supplement: Supplementary file 1 [file curroncol-32-00140-s001.zip › curroncol-3404452-IRB waiver proof.pdf]

ARECCI Ethics Screening Tool Report

arecci.albertainnovates.ca

Form Submitted: 29/01/2025

This does not constitute / represent a formal ethics ruling. Individuals are advised to additionally follow the policies or consult their local ethics authority. ARECCI helps project leads address and mitigate ethical risks by providing decision support tools, training opportunities, and project ethics consultation. albertainnovates.ca/programs/arecci/

Scoring Explanation

Score Result	Risk & Recommended Ethics Review
47 or Greater	**Definitely greater than minimal:** Organization's recognized review process* using ARECCI Ethics Guidelines for Quality Improvement and Evaluation Projects. *Review by a duly constituted group independent of the project team, that is trained to do project ethics reviews and whose decisions are recognized by the organization.
8 - 46	**Somewhat more than minimal:** Second Opinion Review** using ARECCI Ethics Guidelines for Quality Improvement and Evaluation Projects. **Review by an individual trained to do project ethics reviews who has no vested interest in the outcome of the project.
0 - 7	**Minimal:** Project leader uses ARECCI Ethics Guidelines for Quality Improvement and Evaluation Projects

Project Details

Project Title: A Community of Practice: an Interactive, virtual forum for Immuno-oncology Health Professionals

Your score is 0

The project involves Minimal Risk. Use the ARECCI tools to identify and manage risk consistent with local policies.

Type of Project

- Other

Please specify "Other"

Web-based application for information sharing between healthcare professionals

Your Location

Alberta
Canada

Questions that affected your final score:

Preliminary Questions

1. Is there an explicit requirement for review of this project by a Research Ethics Board as part of its funding arrangements?
No

2. Are there any local policies that require this project to undergo review by a Research Ethics Board?
No

3. Does the project involve use of a pharmaceutical device, drug or natural health product under Health Canada Food and Drug Act regulations or guidelines?
No

ALBERTA INNOVATES

arecci
A pRoject Ethics Community Consensus Initiative

Primary Purpose of the Project

4. Is the project designed to test a specific hypothesis or answer a specific quantitative or qualitative question?
No

5. Does the project involve a comparison of control groups?
No

6. Is the project designed to support generalizations that go beyond the particular population the sample is being drawn from?
No

7. Does the project impose any additional burdens on participants beyond what would be normally expected or normally experienced during the course of care, program participation or role expectations?
No

8. Is the primary purpose of the project to produce the kind of results that could be published in a research journal?
No

9. Will project participants also likely be among those who might potentially benefit from the result of the project as it proceeds?
Yes

10. Is the project intended to develop a better practice within your organization or setting?
Yes

11. Would this project still be done at your site even if the results might not be applicable anywhere else?
Yes

12. Does the language used in the project description refer specifically to features of a particular program, organization, or locale, rather than using more general terminology such as rural vs. urban populations?
No

13. Is the current project part of a continuous process of gathering or monitoring data within an organization?
No

ALBERTA INNOVATES

arecci
A pRoject Ethics Community Consensus Initiative

Below are your responses to the Screening Tool questions.

These response options are weighted.

- A ✔ indicates a "Yes" response. Affirmative responses are identified as areas of ethical risk. Please review.
- A ✖ indicates a "No" Response.

Your score indicates that the most probable purpose of your project is Quality Improvement or Program Evaluation. Please proceed to determine the category of risk to your participants.

Risk Filter for Quality Improvement and Evaluation

Does your project involve...

14. Likelihood that a breach of confidentiality could place participants at risk of legal liability, denial of insurance or other damage to financial standing, employability, or reputation?

- **Yes**
- No ✖

15. A real or potential conflict of interest between an investigator and the sponsor of the investigation?

- **Yes**
- No ✖

16. A power relationship between the investigator and participants (e.g., manager/employee, therapist/client, service provider/recipient, teacher/student)?

- **Yes**
- No ✖

17. Questions that collect information about sensitive issues, illegal behaviour, stigmatizing conditions or behaviours, or religious or cultural beliefs or practices?

- **Yes**
- No ✖

18. Inexperienced project leads?

- **Yes**
- No ✖

19. Collection of data through technical procedures or diagnostic tools routinely employed in the setting?

- **Yes**

- No

20. The use of tests, surveys, interviews, oral history, focus groups, or observation of public behaviour where the participants can be directly or indirectly identified through the information recorded?
- **Yes**
- No

21. Collection of data from voice, video, digital or image recordings?
- **Yes**
- No

22. Personally identifiable data, documents, records or specimens originally collected solely for purposes not related to the current study?
- **Yes**
- No

23. Special populations or any individuals or groups in a socially vulnerable position?
- **Yes**
- No

24. An original or novel process for which it would be difficult to estimate a balance of risk and benefit in advance?
- **Yes**
- No

25. Risks of breaching the confidentiality of any individual's personal information beyond that experienced in the provision of routine service or day-to-day life?
- **Yes**
- No

26. A person who does not normally have access to participant records and whose use of records is for a secondary purpose?
- **Yes**
- No

27. Any significant departure from the routine care, program, or service provided to participants or the gathering of information about participants beyond that normally collected?
- **Yes**
- No

28. Risks or burdens for participants which are beyond what would be experienced in routine care or beyond what a reasonable person might expect in day-to-day interactions?
- **Yes**
- No

29. Questions or procedures that might cause participants psychological distress, discomfort or anxiety beyond

what a reasonable person might expect in day to day interactions?

- **Yes**
- No ✖

30. Intended deception or intended incomplete disclosure of the nature of the investigation?

- **Yes**
- No ✖

31. Evaluation of the safety and effectiveness of a mechanical device, drug, or natural health product?

- **Yes**
- No ✖

32. Clinical studies on a device, drug or natural health product where Health Canada review and approval is not required?

- **Yes**
- No ✖

33. Therapeutic procedures that are themselves known to pose considerable risks of harm?

- **Yes**
- No ✖

34. Any procedures related to anesthetics, sedation, or any alteration of medication that is not normally part of participant care or health?

- **Yes**
- No ✖

35. Non-invasive procedures beyond what is normally required for participant care?

- **Yes**
- No ✖
