# Supplementary material for: Enhancing Care Through a Virtual Canadian Community of Practice for Managing Immune-Related Adverse Events
Source: Curr Oncol. 2025 Feb 27;32(3):140. doi: 10.3390/curroncol32030140 (PMC11941491; doi:10.3390/curroncol32030140)
Supplement: Supplementary file 1 [file curroncol-32-00140-s001.zip › Table S1.pdf]

Supplementary Table S1

Table S1. ONCOassist app key features		
**Feature**	**Description**	**Examples**
**Formulas**	Over 20 interactive formulas including offline access to enable clinicians to make the necessary calculations at point of care	Body surface area / chemotherapy dose calculator to adjust the chemo dosage if a patient loses or gains weight since the last prescribed dosage
**AJCC TNM Staging:**	Enables an easy and quick feature to help clinicians in their cancer reporting and classification	Tumor size, lymph nodes affected, metastases.
**Adjuvant tools**	Can be used to get a 5 and 10-year overall survival of patients with and without chemotherapy in an adjuvant setting, this helps them inform patients as to why they may or may not be prescribing chemotherapy	Prediction algorithm estimating survival rates for breast/lung/colon/GIST cancer based upon risk factors and treatment
**Prognostic Scores**	Prognostic scores: over 14 prognostic scores enabling clinicians to get the scores they are looking for based on a few questions and patient characteristics	Predicting survival in patients with metastatic renal cell carcinoma
**Toxicity Grading**	A set of criteria for the standardized classification of adverse effects of drugs used in cancer therapy.	CTC AE version 4 and 5
**IO Toxicity Tool**	Integrates guideline recommendations on the management of immune-related adverse events from oncology societies	-
**Drug Info**	Gives users access to a comprehensive list of oncology specific drugs information allowing them to jump in and out of specific sections quickly	-
**Drug Interaction checker:**	Enables users to quickly search combinations of drug interactions to identify if they are safe to use.	-
**ONCOnews:**	Allows easy access to most up to date news and information in the field of oncology, personalized based on the user's specialist interests.	-
**CoP on irAEs**	Available to Canadian ONCOassist users only	
Abbreviations: AJCC: American Joint Commission on Cancer; CoP: community of practice, CTC AE: Common Terminology Criteria for Adverse Events, irAE: immune related adverse event, TNM: tumor, regional lymph nodes, distant metastasis,		
